# Case Report: Laparoscopic hepatectomy in an elderly patient with major comorbidities

**DOI:** 10.12688/f1000research.12078.2

**Published:** 2017-12-18

**Authors:** Georgios C. Sotiropoulos, Nikolaos Machairas, Ioannis D. Kostakis

**Affiliations:** 1 Second Department of Propedeutic Surgery, Laiko General Hospital, National and Kapodistrian University of Athens, Medical School, Athens, Greece

**Keywords:** hepatectomy, liver resection, laparoscopic, elderly, geriatric, comorbidity

## Abstract

Surgeons have been hesitant to proceed to hepatectomy in elderly patients, due to the higher rate of comorbidities and the reduced reserves. An 81-year-old male with hepatocellular carcinoma in the segment VI of the liver and several major cardiovascular, pulmonary and metabolic comorbid illnesses was referred to our department for treatment. He underwent transarterial chemoembolization of the liver tumor and afterwards he underwent laparoscopic resection of the hepatic segment VI, with an uneventful postoperative course. This case indicates that laparoscopic liver resections could be applied even to elderly patients with major comorbidities after optimization of their medical status.

## Introduction

Liver resection is the treatment of choice for many liver tumors. However, liver resections, and especially major hepatectomies, have been associated with several complications and the presence of comorbid illnesses has been related to increased postoperative morbidity and mortality ^1^. Surgeons have been hesitant to proceed to hepatectomy in elderly patients, due to the higher rate of comorbidities and the reduced hepatic, cardiac, pulmonary and renal reserve, which render them more susceptible to complications. However, there are several attempts towards the adaptation of liver resections for elderly patients that have been reported in the literature, with good outcomes ^2^. We report the case of an 81-year-old male with hepatocellular carcinoma and several comorbid illnesses who underwent laparoscopic liver segmentectomy in our department.

## Case report

**Figure 1.**
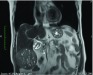
Magnetic resonance imaging of the giant liver lesion.

An 81-year-old male patient with deteriorating right subcostal pain and high values of serum gamma-glutamyl transpeptidase and alkaline phosphatase underwent an abdominal ultrasound scan, which revealed the presence of a heterogeneous tumor 10cm in diameter, located in the right hepatic lobe, along with mild steatosis of the liver. The patient underwent an abdominal and thoracic computed tomography and an abdominal magnetic resonance imaging. They showed a heterogeneous tumor in the hepatic segment VI, which presented intense arterial uptake of the intravenous contrast followed by quick venous washout, without any sites suspicious for metastases ( Figure 1). Additionally, he underwent an ultrasound-guided biopsy of the mass, which revealed the presence of a moderately differentiated hepatocellular carcinoma (HCC). Serum levels of alpha-fetoprotein (AFP) were within normal range. The patient’s medical history included arterial hypertension, type 2 diabetes mellitus, atrial fibrillation and severe chronic obstructive pulmonary disease (COPD) with chronic productive cough, rendering him a patient with an ASA (American Society of Anesthesiologists) score 3, but no viral hepatitis, cirrhosis or any other liver pathology, apart from mild liver steatosis. Moreover, there was no evidence of renal disease (creatinine serum levels: 0.9 mg/dl, urea serum levels: 25 mg/dl) or anemia (hematocrit: 42.1%, hemoglobin: 14.1 g/dl). The patient’s body mass index (BMI) was 26, he was functionally independent and he was capable of physical activity of light to moderate intensity (Metabolic Equivalent of Task: 3) ^3, 4^.

The patient was referred to our department for treatment. Blood gas analysis revealed the following parameters: pO _2_: 58mmHg, pCO _2_: 45mmHg, HCO _3_: 26mEq/L, pH: 7.38, while spirometry showed FEV1: 47% predicted and FEV1/FVC: 55% predicted, revealing the presence of severe COPD ^5^. The patient received both pulmonary and anesthesiological consultation, and he was treated with daily bronchodilations and respiratory physiotherapy. The patient initially underwent a transarterial chemoembolization of the HCC as a bridging treatment to operation. He was followed-up in an outpatient basis for about a month. His blood gas analysis showed a notable improvement: pO _2_: 75mmHg, pCO _2_: 39mmHg, HCO _3_: 24mEq/L, pH: 7.41, as well as the results of spirometry (FEV1: 60% predicted, FEV1/FVC: 66% predicted), along with amelioration of productive cough. The patient was admitted to our hospital and he underwent laparoscopic resection of the hepatic segment VI, which contained the tumor, along with laparoscopic cholecystectomy. The postoperative course was uneventful and the patient was discharged on the 4th postoperative day.

**Figure 2.**
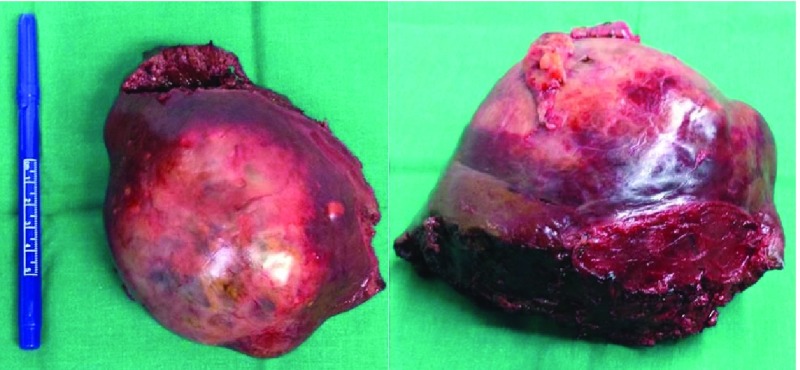
Resected liver lesion.

The histopathological examination of the surgical specimen showed that the tumor corresponded to a moderately differentiated hepatocellular carcinoma, grade II and III in the Edmondson-Steiner grading scale, with infiltration, but not disruption of Glisson’s capsule, and without infiltration of blood vessels (pT1 tumor) ( Figure 2). The resection margins were tumor-free. The histopathological examination also confirmed the mild liver steatosis that the abdominal ultrasound had indicated. The patient remains in good general condition without evidence of tumor recurrence 30 months after the operation.

## Discussion

Elderly patients frequently have a fragile health, as a result of many kinds of comorbidities that present at their age that are associated with reduced reserves. The arising higher susceptibility of elderly patients to complications makes surgeons more reluctant to proceed to major operations in these patients ^6^. Therefore, liver resections for old patients, and especially major hepatectomies, have been adopted with delay ^2^.

However, several studies have addressed feasibility, efficacy and safety of liver resections in elderly patients. Although there are various cut-off points for the definition of elderly patients among these studies, most of them use 70 ^7^ or 75 years ^8– 10^ of age as a threshold to define older patients. All types of liver resections have been reported for patients with advanced age, from wedge resections and segmentectomies up to hemihepatectomies. Many studies have reported that there is no actual advantage regarding morbidity and mortality of younger over older patients undergoing liver resection, if older patients are considered fit enough to undergo the procedure ^10^. Nevertheless, there are also several studies reporting an increased rate of postoperative morbidity and/or mortality for older patients than younger ones ^7– 9^.

Apart from the age as an independent predictor of postoperative outcomes, the existence of comorbidities has been evaluated as an important factor of worse postoperative results. Several studies have shown that patients undergoing liver resection who suffer from arterial hypertension, diabetes mellitus, arrhythmias, coronary disease, heart failure, chronic obstructive pulmonary disease, renal disease, liver cirrhosis, stroke and/or other major comorbidities have increased postoperative morbidity and/or mortality in comparison with the patients with only minor or without any comorbid diseases. An ASA score of 3 or greater has been associated with higher rates of postoperative complications and worse outcomes in general ^7, 8^.

There are only a few series reporting laparoscopic liver resections in elderly patients. When older patients (older than 70 or 75 years of age) with liver pathology undergoing laparoscopic liver resection were compared to younger ones, no significant differences were detected with regards to postoperative morbidity and mortality ^11, 12^. Furthermore, when laparoscopic and open hepatectomies were compared in elderly patients, there was no agreement concerning postoperative complications, with some authors reporting decreased rates in the case of laparoscopic hepatectomies ^12^, whereas other authors suggested that there is no actual difference between the two approaches ^13^. However, it is accepted that laparoscopic procedures have the advantage of less blood loss and shorter hospital stay in elderly patients ^12, 13^.

Our patient aged 81 years and had some major comorbidities. However, the careful therapeutic planning with the optimization of his pulmonary status and the careful selection of the exact type of liver resection rendered the patient able to undergo the laparoscopic segmentecotmy with an uneventful postoperative course. In conclusion, laparoscopic liver resections could be applied even to elderly patients with major comorbidities after optimization of their medical status.

## Consent

Written informed consent for publication of clinical details and/or clinical images was obtained from the patient.
