# Supplementary material for: Case Report: Laparoscopic hepatectomy in an elderly patient with major comorbidities
Source: F1000Res. 2017 Dec 18;6:1286. Originally published 2017 Jul 31. [Version 2] doi: 10.12688/f1000research.12078.2 (PMC5747337; doi:10.12688/f1000research.12078.2)
Supplement: Peer review history

# Peer review: Referee response for version 2

**DOI:** 10.5256/f1000research.14671.r29101

**Published:** 2017-12-22

**Authors:** Troy S. Wildes

**Affiliations:** 1 Department of Anesthesia, Washington University School of Medicine (WUSM), St. Louis, MO, USA; 2 Center for Preoperative Assessment and Planning, Barnes-Jewish Hospital (BJH) and Washington University School of Medicine (WUSM), St. Louis, MO, USA

The authors have fully addressed my initial reservations regarding their manuscript. They have provided key additional information regarding the patient's comorbidities, tests, and function that are useful for gauging perioperative risks.

I have read this submission. I believe that I have an appropriate level of expertise to confirm that it is of an acceptable scientific standard.

# Peer review: Referee response for version 1

**DOI:** 10.5256/f1000research.13067.r28236

**Published:** 2017-11-30

**Authors:** Troy S. Wildes

**Affiliations:** 1 Department of Anesthesia, Washington University School of Medicine (WUSM), St. Louis, MO, USA; 2 Center for Preoperative Assessment and Planning, Barnes-Jewish Hospital (BJH) and Washington University School of Medicine (WUSM), St. Louis, MO, USA

The authors provide an interesting case report of an aged individual with significant comorbidities undergoing an intermediate risk surgical procedure.

 The operative and optimization pathway are discussed in appropriate detail. The authors are also to be commended for including the patient's health status 30 months after the surgery, as such information is clearly relevant to surgical decision-making in patients with significant comorbidities.

 The report would benefit from the following additional details and suggested edits:

1. The case presented does not justify the statement "In conclusion, laparoscopic liver resections are safe and feasible in older patients, even when major comorbidities are present, provided that there is a careful therapeutic planning." Describing a medical practice as safe implies a very low event rate, which cannot be demonstrated with a single successful patient outcome.
2. The patient's medical comorbidities are listed. However, important functional details such as the patient's functional independence, metabolic functional capacity, and COPD symptom burden are not described. Such details would be critically informative of the comorbidity disease burden, frailty status, and anticipated complication rate of the patient. Additionally, inclusion of other key diagnostic information, such as serum creatinine and hemoglobin, would be relevant along with the above information for predicting pulmonary and acute kidney injury risk. Without inclusion of all of this information, it is difficult for the reader to truly appreciate the risk level of this patient.

I have read this submission. I believe that I have an appropriate level of expertise to confirm that it is of an acceptable scientific standard, however I have significant reservations, as outlined above.

# Response

**Published:** 2017-12-13

**Authors:** Ioannis Kostakis

**Affiliations:** Second Department of Propedeutic Surgery, “Laiko” General Hospital, National and Kapodistrian University of Athens, Medical School, Greece

**Comment 1**

The case presented does not justify the statement "In conclusion, laparoscopic liver resections are safe and feasible in older patients, even when major comorbidities are present, provided that there is a careful therapeutic planning." Describing a medical practice as safe implies a very low event rate, which cannot be demonstrated with a single successful patient outcome.

**Answer**

            We removed this sentence and we replaced it with the following sentence: “Laparoscopic liver resections could be applied even to elderly patients with major comorbidities after optimization of their medical status”.

**Comment 2**

The patient's medical comorbidities are listed. However, important functional details such as the patient's functional independence, metabolic functional capacity, and COPD symptom burden are not described. Such details would be critically informative of the comorbidity disease burden, frailty status, and anticipated complication rate of the patient. Additionally, inclusion of other key diagnostic information, such as serum creatinine and hemoglobin, would be relevant along with the above information for predicting pulmonary and acute kidney injury risk. Without inclusion of all of this information, it is difficult for the reader to truly appreciate the risk level of this patient.

**Answer**

            We added data regarding patient’s functional independence, ability for physical activity, creatinine and urea serum levels, hematocrit, COPD symptoms and results of spirometry.

# Peer review: Referee response for version 1

**DOI:** 10.5256/f1000research.13067.r27703

**Published:** 2017-11-20

**Authors:** Jun Li

**Affiliations:** 1 Department of Hepatobiliary and Transplant Surgery, University Medical Center Hamburg-Eppendorf, Hamburg, Germany

The case report by Sotiropoulos *et al* did show that combining the minimal invasive approach and preoperative  optimal management of the comorbidities could make liver resection in very elderly patient safe. The preoperative TACE not only served as a back-up treatment for the large HCC, but also might reduce the risk of metastasis.

I have read this submission. I believe that I have an appropriate level of expertise to confirm that it is of an acceptable scientific standard.

# Response

**Published:** 2017-12-13

**Authors:** Ioannis Kostakis

**Affiliations:** Second Department of Propedeutic Surgery, “Laiko” General Hospital, National and Kapodistrian University of Athens, Medical School, Greece

We would like to thank the reviewer for his kind comments.
